# Multiple giant cutaneous metastasis and ileal intussusception from an unknown melanoma: A case report

**DOI:** 10.3892/mi.2024.175

**Published:** 2024-07-11

**Authors:** Alberto Vilar, Eduardo Serrano, Philip Brabyn, Manuel Mariano Diez, Alberto Gutierrez

**Affiliations:** 1 Department of General and Digestive Surgery, Hospital Universitario Príncipe de Asturias, 28805 Alcalá de Henares, Spain; 2 Department of Head and Neck Surgery, Hospital Universitario Niño Jesús, 28009 Madrid, Spain

**Keywords:** ileal intussusception, melanoma, metastatic melanoma, cutaneous metastasis

## Abstract

Intestinal intussusception is one of the most common causes of intestinal obstruction in children; however, the incidence in adults is lower, and is caused by tumors in the majority of cases. Melanoma of the gastrointestinal tract is relatively rare, with only a small number of cases having been reported. The majority of cases occur as metastasis from cutaneous primary lesions, and the small bowel is the most common location of melanoma metastases in the gastrointestinal tract. The present study describes the case of a 47-year-old male patient with multiple soft tissue tumors, the largest one located in the left gluteal region, measuring 14x15x20 cm. This tumor was biopsied and a differential diagnosis was made between clear cell sarcoma and melanoma. The patient was evaluated by a dermatologist, without identifying any skin lesions compatible with cutaneous melanomas and was admitted to the emergency room due to an ileo-ileal intussusception. The results of the pathological analysis confirmed the final diagnosis of melanoma. On the whole, these lesions are usually diagnosed with the onset of symptoms, presenting an ominous prognosis.

## Introduction

Small bowel obstructions are common, being secondary in most cases to adhesions from previous surgeries, followed by abdominal hernias and intestinal tumors. Intestinal intussusception is one of the most common causes of intestinal obstruction in children; however, the incidence in adults decreases, caused by tumors in most cases. Small bowel tumors involve only 5% of all gastrointestinal malignancies, of which 44% are carcinoid tumors, 33% are adenocarcinomas, 17% are stromal tumors and 8% lymphomas. Melanoma of the gastrointestinal tract is relatively rare, with only a few cases having been reported (1).

The majority of cases occur as metastasis from cutaneous primary lesions, and the small bowel is the most common location of melanoma metastases in the gastrointestinal tract (2). The majority of melanomas are cutaneous in origin; however, ~3% of cases occur as metastases with an unknown primary source even following thorough investigations (3). In some patients, it is possible to describe a previous suspicious naevus, which spontaneously regresses (4). Patients with melanoma of unknown primary usually present with locoregional melanoma metastases in the (sub)cutis, soft tissue, and/or lymph nodes (i.e., stage III disease) or with distant metastases including visceral metastases (i.e., stage IV disease) (5). Metastases of malignant melanoma also can lead to soft tissue sarcomas (6).

## Case report

**Figure 1:**
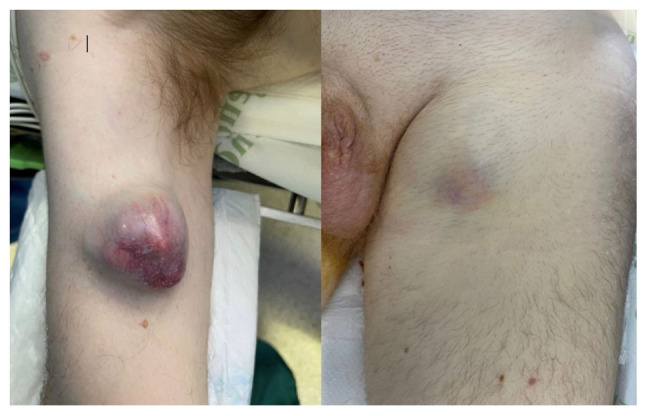
Soft tissue tumors in the right arm (left panel) and left inguinal site (right panel).

The present study describes the case of a 47-year-old male patient without any previous medical conditions, who visited Príncipe de Asturias University Hospital (Alcalá de Henares, Spain) for multiple soft tissue tumors, the largest one being located in the left gluteal region, measuring 14x15x20 cm. Other lesions were located in the left inguinal region, measuring 8x5x5 cm and in the right arm, measuring 5 cm, presenting with a rapid growth pattern during the previous month (Fig. 1).

**Figure 2:**
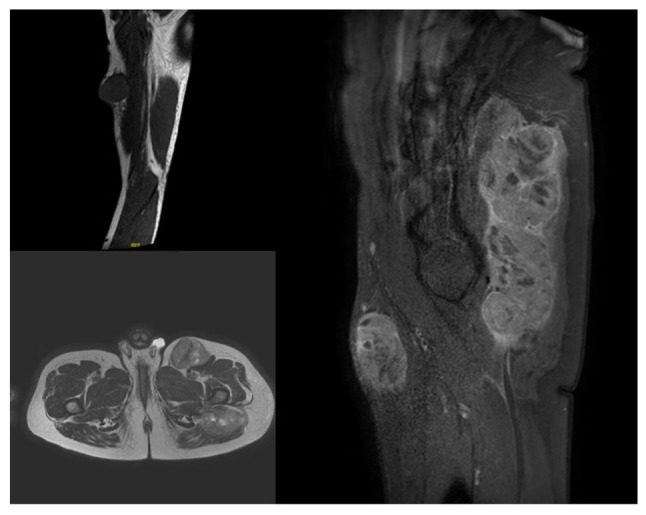
Magnetic resonance imaging scan illustrating three tumors, on the right arm (top left panel), left inguinal site (bottom left panel) and left gluteal region (right panel).

**Figure 3:**
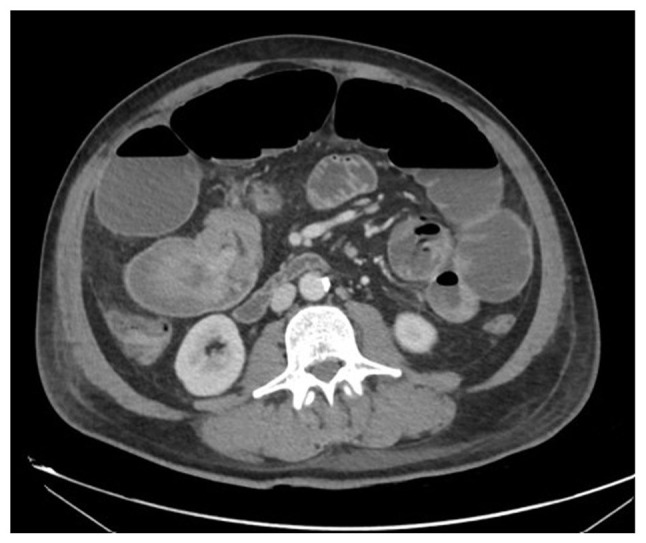
Computed tomography scan illustrating an ileo-ileal intussusception.

A magnetic resonance imaging scan was performed, identifying the three masses (Fig. 2), and the gluteal tumor was biopsied reporting a differential diagnosis between clear cell sarcoma and melanoma. Before other tests could be performed to confirm the diagnosis, the patient was admitted to the emergency room for an intestinal obstruction. A computed tomography scan was performed, which revealed an ileo-ileal intussusception (Fig. 3). Pulmonary nodules and bilateral liver lesions were also described according to metastatic disease.

**Figure 4:**
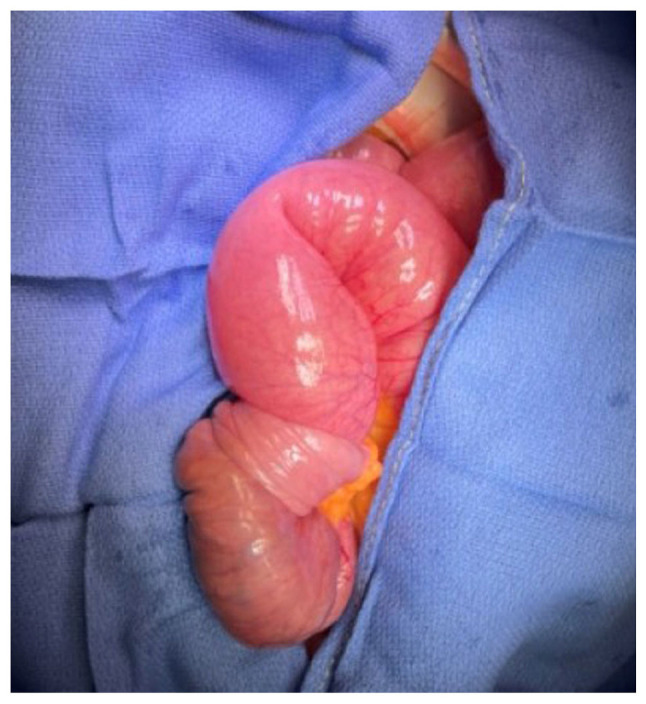
Image illustrating an intraoperative invaginating tumor in the proximal ileum.

The patient was evaluated by a dermatologist, without identifying any skin lesions compatible with cutaneous melanomas and only a small nevus was signed to biopsy on the left leg. An urgent laparotomy was performed, in which abdominal fluid was observed (sent for cytological analysis), as well as an invaginating tumor in the proximal ileum (Fig. 4). An ileal resection and a side-to-side anastomosis were performed. Hepatic nodules, as well as peritoneal implants in the greater omentum and meso-jejunum were identified. The greater omentum and meso-jejunum implants were sent for a pathological anatomical analysis, together with the tumor of the right arm and the nevus signed by dermatology on the left leg.

The results of the pathological analysis revealed that the nevus in the left leg was a melanocytic nevus. The lesion in the right arm, the cytology of the peritoneal fluid, the peritoneal implants, the omentum, as well as the lesion in the small intestine, corresponded to an undifferentiated malignant neoplasm that, with the immunohistochemical analysis (performed by another laboratory), exhibited positivity for vimentin, S100, HMB-45, SOX 10, Melan-A, and negativity for CD 117, DOG 1, muscle actin, CK7, CK20, synaptophysin, desmin and CD56.

The presence of the EWSRI I t(12;22)(ql3;ql2)] gene rearrangement present in 90% of clear cell sarcoma cases was ruled out, thus confirming the final diagnosis of melanoma.

The patient had a satisfactory immediate post-operative period and was transferred to the oncological department to commence treatment with chemotherapy. However, the patient succumbed during the hospital stay due to a respiratory infection on the 14th post-operative day.

## Discussion

Intestinal intussusception is common among children, but unusual in adults, where it is associated with an underlying malignant process. Intussusception secondary to melanoma is detected in 2-5% of patients with a history of melanoma, and it is possible to find such intestinal metastases up to 10 years after the skin lesion (7).

Small bowel melanoma is a rare entity, with the majority of cases being secondary to metastasis of a primary tumor. The small bowel is the location where a primary cutaneous melanoma metastasizes most frequently to the gastrointestinal tract, due to the rich vascular support of the splanchnic territory. There are a limited number of published cases of primary melanoma in this location, due to the absence of melanocytes (1). It is critical to make a differential diagnosis between primary melanoma of the gastrointestinal tract and metastasis as the prognosis is worse for primary intestinal melanomas, which tend to grow at a more rapid rate and are more aggressive (8).

When the skin melanoma is not identified, it is mandatory to perform a colonoscopy (to rule out an anorectal melanoma), gastroscopy (to rule out an oropharynx, esophagus or stomach melanoma), as well as a fundoscopic examination (choroid melanoma) (9). However, in up to 26% of cases of intestinal melanomas, no extraintestinal primary lesion can be identiﬁed. In such circumstances, the spontaneous regression of the primary site may explain the lack of a primary melanoma. In the case in the present study, it was not possible to perform these tests as the patient suffered an intestinal obstruction and ultimately succumbed during the hospitalization period due to a respiratory infection on the 14th post-operative day; however, the absence of any other lesion led to the conclusion that it was a small bowel melanoma or soft tissue and visceral metastasis from an unknown melanoma.

This type of metastasis is usually silent until the moment of diagnosis. The majority of patients with metastatic intestinal melanoma are asymptomatic and only 1-4% of metastases to the gastrointestinal tract are detected before death (10). In the case in the present study, the patient presented with multiple giant soft tissue tumors and an ileal intussusception. There are previous reports of soft tissue metastasis and ileal metastasis (6); however, the patient in the present study suffered both site metastasis.

The management of such cases consists of surgery to solve the acute obstruction. In cases where metastatic disease is limited and a resection is possible, surgery can be considered. Even when a R0-status and a curative surgery cannot be achieved or there is recurrent disease, tumor resection is recommended to relieve symptoms or avoid future complications (11). Systemic treatment is not standardized; however, systemic therapy with immunotherapy, chemotherapy or molecular targeted therapy, such as vemurafenib, ipilimumab, pembrolizumab or imatinib, are other options (12). They can also be useful as a palliative treatment in metastatic intestinal melanoma; however, role remains unclear (13).

In conclusion, gastrointestinal tract melanoma is a rare entity, involving metastatic lesions in the majority of cases. These lesions are usually diagnosed with the onset of symptoms, presenting an ominous prognosis. In cases of bowel obstruction, a surgical resection is required.

## Data Availability

The datasets used and/or analyzed during the current study are available from the corresponding author on reasonable request.
